# Exogenous dietary lysozyme improves the growth performance and gut microbiota in broiler chickens targeting the antioxidant and non-specific immunity mRNA expression

**DOI:** 10.1371/journal.pone.0185153

**Published:** 2017-10-23

**Authors:** Mervat A. Abdel-Latif, Ali H. El-Far, Ahmed R. Elbestawy, Rania Ghanem, Shaker A. Mousa, Hatem S. Abd El-Hamid

**Affiliations:** 1 Department of Nutrition and Veterinary Clinical Nutrition, Faculty of Veterinary Medicine, Damanhour University, Damanhour, AlBeheira, Egypt; 2 Department of Biochemistry, Faculty of Veterinary Medicine, Damanhour University, Damanhour, AlBeheira, Egypt; 3 Department of Poultry Diseases, Faculty of Veterinary Medicine, Damanhour University, Damanhour, AlBeheira, Egypt; 4 Animal Health Research Institute, Mansoura Laboratory, Mansoura, Egypt; 5 Pharmaceutical Research Institute, Albany College of Pharmacy and Health Sciences, Albany, New York, United States of America; 6 Department of Poultry Diseases, Faculty of Veterinary Medicine, Damanhour University, Damanhour, AlBeheira, Egypt; Leibniz-Institut fur Pflanzengenetik und Kulturpflanzenforschung Gatersleben, GERMANY

## Abstract

Supplementation of exogenous enzymes in chickens has been widely practiced, yet mechanisms responsible are not fully delineated. To investigate the effects of the dietary lysozyme on the growth performance and immunity of broiler chickens, a total of 120 one-day-old Ross 308 chicks were randomly allocated into four groups, each having three replicates (30 birds/group). The chicks were fed the starter (1–21 d) and grower (22–35 d) diets supplemented with 0 (control), 70 (LYZ70), 90 (LYZ90) and 120 (LYZ120) g of lysozyme 10%^®^ per ton of basal diet for five weeks. The results revealed significant improvement in the growth performance and gut environment. There were significant decreases (*P* < 0.05 or 0.01) in the harmful fecal *Coliform* and *Clostridia* and an increase (*P* ˂ 0.05) in the beneficial *Lactobacillus* in the lysozyme-supplemented groups, especially in LYZ90. Moreover, the mRNA expressions of Cu, Zn-superoxide dismutase (*SOD1*), glutathione peroxidase (*GSH-Px*), interferon-gamma (*IFN-γ*), interleukin-10 (*IL-10*), and interleukin-18 (*IL-18*) were upregulated in response to lysozyme supplementation. In comparison to control, LYZ90 fed birds had a significant increase (*P <* 0.01) in the *GSH-Px* gene expression that enhances the antioxidant status of the gut. Expression of the biomarkers involved in the gut non-specific immunity indicated significant increases in the mRNA expression of *INF-γ* (*P <* 0.001), *IL-10* (*P <* 0.001), and *IL-18* (*P <* 0.05) in LYZ90 group. Also, serum globulin levels were significantly elevated (*P* ˂ 0.05) in lysozyme-supplemented groups. Histologically, the intestinal villi length and crypts depth were also enhanced (*P* ˂ 0.05) by dietary lysozyme supplementation. In conclusion, supplementation of broiler chickens with exogenous lysozyme, especially at 90 g of lysozyme per ton of basal diet dose rate, improved the growth performance, gut antioxidant status, and nonspecific immunity of broiler chickens.

## Introduction

The intestinal microbiota has a great impact on host health, while its disturbances have been associated with various diseases [1]. Gut microbial communities improve the animal health through the synthesis of vitamins, food digestion, and immunity. Antibiotics are the most potent factor leading to disturbances in the gut microbial community in human and animal [2]. Antibiotics have been used for many years in animal production. They can be used as growth promoters for broiler chickens because they control the growth of both the Gram-positive and Gram-negative bacteria in the gut [3]. Currently, the European Union has banned antibiotics’ supplementations in poultry flocks because of the potential human pathogens’ resistance toward certain antibiotics [4,5]. Thus, the poultry industry is increasingly in need of non-antibiotic alternatives to improve the gut health in commercial broiler chickens. Farmers have conveyed the use of alternative dietary supplements including probiotics, prebiotics, herbs, and exogenous enzymes instead of antibiotics [6–8].

There are four distinct categories of enzyme feed additives commercially available for use: (1) microbial phytases, (2) glycanases targeting viscous cereals such as wheat and barley, (3) enzymes targeting non-viscous cereals like corn and sorghum, and (4) enzymes targeting non-cereals such as soybean meal and grain legumes [9]. The combined application of enzymes may result in synergistic effects on nutrient utilization and animal performance [10].

Lysozyme (EC 3.2.1.17) is a natural antimicrobial protein considered to be an important component of the innate immune system [11]. It exerts bacteriolytic activity by hydrolyzing the β-1,4-glycosidic linkage between N-acetylmuramic acid and N-acetyl glucosamine of bacterial cell wall, mainly against many Gram-positive bacteria [12]. Because of its abundance in egg white, lysozyme is commercially extracted from eggs and has been applied as a natural food preservative and a therapeutic drug for humans [13]. Also, the *in vivo* intraperitoneal administration of lysozyme decreases the pathology resulting from *Klebsiella pneumoniae* in mice [14]. Thus, it provides protection against bacterial diseases. Results from *in vitro* studies stated that the antimicrobial activity of exogenous lysozyme against *Clostridium perfringens* type A in broiler chickens was associated with necrotic enteritis. It was reported that lysozyme could control *C*. *perfringens* type A [15,16]. Also, exogenous lysozyme reduced the number of *C*. *perfringens* in the ileum of broiler chickens and prevented the intestinal lesions when chickens were experimentally infected by *C*. *perfringens* orally [11]. Changes in microbiota may reduce the adverse effect of anti-nutritional factors in feeds that modulate microbiota and immune function, improve the intestinal morphology, and decrease the gut oxidative stress [17].

There are a limited number of studies focused on the effect of different levels of exogenous lysozyme supplementation on broiler chickens’ performance and gut health. Therefore, the current study was conducted to investigate the effect of the dietary supplementation of exogenous lysozyme on the growth performance and gut health in broiler chickens.

## Materials and methods

### Ethics statement

This study was carried out in strict accordance with the recommendations of the Committee on the Ethics of Animal Experiments of Damanhour University, Egypt. All procedures and experiments complied with the guidelines and were approved by the Local Ethic Commission of the Animal Experiments of Damanhour University with respect to animal experimentation and care of animals under study. All efforts were made to minimize suffering.

### Birds and housing

In total, 120 one-day-old Ross 308 chicks were obtained from a commercial hatchery and reared on wire-floored cages of the same dimensions with the same number of nipple drinkers and feed hoppers.

The incubation temperature of 32°C was gradually decreased to 26°C by the 3rd week of age, and the chicks were exposed to 23 h of light.

### Diet

A corn-soybean based basal diet was formulated for the starter phase (1–21 d) (23% CP & 3094 Kcal/kg diet) and the grower phase (22–35 d) (20% CP & 3142 Kcal/kg diet), which could meet the requirements of broiler chickens for respective phases as per National Research Council (NRC) recommendations [18]. The ingredients and nutrient composition of the basal diet were analyzed according to AOAC [19] and are shown in Table 1. No antibiotic was supplemented during the experimental period. The birds consumed feed *ad libitum* and a record of body weight and feed intake was maintained weekly during the experiment. Water was made available at all the times and birds were observed daily.

**Table 1:** Composition of the experimental starter and finisher diets.

**Ingredient**	**Starter (%)**	**Grower (%)**
**Corn**	53.71	61.92
**Soybean meal (crude protein 44%)**	33.42	28.05
**Corn gluten (60%)**	5.22	3.20
**Corn oil**	3.32	2.94
**Limestone**	1.28	1.15
**Dicalcium phosphate**	1.84	1.68
L- lysine*	0.12	0.14
DL-methionine**	0.39	0.22
Vitamins and minerals premix***	0.30	0.30
**NaCl**	0.40	0.40
**Total**	100	100
**Analyzed and calculated composition**	**Starter**	**Grower**
**Metabolizable energy**^**¶**^ **(Kcal/kg diet)**	3094	3142
**Crude protein %**	23	20
**Calorie/protein ratio**	134.50	157.10
**Lysine %**	1.25	1.11
**Methionine %**	0.80	0.58
**Calcium %**	1.00	0.90
**Available phosphorous %**	0.49	0.45
**NaCl %**	0.15	0.15

* 99% feed grade.

** 99% feed grade (Ningbo Haixin Co., Zhejiang, China).

*** Hero mix^®^ (Hero pharm, Cairo, Egypt). Composition (per 3 kg): vitamin A 12000000 IU, vitamin D3 2500000 IU, vitamin E 10000 mg, vitamin K3 2000 mg, vitamin B1 1000 mg, vitamin B2 5000 mg, vitamin B6 1500 mg, vitamin B12 10 mg, niacin 30000 mg, biotin 50 mg, folic acid 1000 mg, pantothenic acid 10000 mg, manganese 60000 mg, zinc 50000 mg, iron 30000 mg, copper 4000 mg, iodine 300 mg, selenium 100 mg, and cobalt 100 mg.

^**¶**^ Calculated per NRC [18].

### Study design

The birds were randomly distributed into four groups of mixed sexes (30 birds per group), each constituting of three replicates. The control group was fed the commercial basal diet and the lysozyme-supplemented groups were supplemented with basal diet containing lysozyme 10%^®^ (Nan Chang Lifeng Industry and Trading Co., Ltd., Jiangxi, China) at the dose rates of 70 g, 90g, and 120g per ton of basal diet for LYZ70, LYZ90, and LYZ120, respectively. Each 1 kg of lysozyme 10%^®^ contained 100 g of lysozyme, 50 g of glycine, 10 g of asparagic acid, 8 g of water, and 832 g glucose.

### Vaccination

Broiler chickens in all cages were vaccinated according to the following schedule: Newcastle disease and infectious bronchitis vaccine (Nobilis^®^ Ma5+Clone 30, Intervet, Boxmeer, Netherlands) at 7 d, Gumboro Intermediate Plus (Bursine Plus^®^ vaccine, Zoetis, NJ, USA) at 14 d, and LaSota (Nobilis^®^ ND LaSota, Intervet) vaccine at 18 d of age. All vaccines were administrated via eye drops.

### Samples collection

Fecal samples from each group (*n* = 10) were taken at 21 and 35 d of age and transferred to buffered peptone water (BPW) (Oxoid, Hampshire, UK) that immediately was used for the intestinal bacterial counts.

Blood samples (*n* = 10) were collected from wing vein at 21 and 35 d of age without anticoagulant for serum separation. Samples were centrifuged at 1435 × g for 5 min at 4°C to obtain clear sera for HI test against NDV and biochemical analyses.

At the end of the experiment, 35 d, intestinal samples (*n* = 10) from each group were taken under anesthesia, whereby each bird was intravenously injected with sodium pentobarbital (50 mg/kg) and immediately necropsied. Samples of 1 cm were taken from ileum (5 cm from Meckel’s diverticulum) and washed immediately with physiological saline (0.9% NaCl). Each sample was kept in an Eppendorf tube and immersed instantly in liquid nitrogen for mRNA expression. Additionally, samples of 3 cm were obtained from jejunum (1 cm cut from the midpoint), washed with saline, and kept in 10% neutral buffered formalin for 24 h for histological examination [20].

### Intestinal bacterial counts

Ten-fold dilutions (10^−1^ to 10^−7^) of each sample were performed with BPW and directly inoculated on MacConkey’s agar (Table 2) for total *Coliform* counting and incubated aerobically at 37°C for 24 h. All red colonies within the range of 15–150 μm were selected for counting [21]. *C*. *perfringens* were subcultured on Perfringens agar base (Oxoid; Table 3) mixed with 400 mg of D-cycloserine per liter by the dilutions from 10^−1^ to 10^−7^ and incubated anaerobically at 37°C using gas generating kits (Oxoid) for 48 h. Plates with black colonies within the range of 25–250 μm were counted [22].

**Table 2:** Composition of MacConkey’s agar.

Ingredients	Amount (g)
**Peptone (Pancreatic digest of gelatin) **	17.0 g
**Proteose peptone (meat and casein)**	3.0 g
**Lactose monohydrate **	10.0 g
**Bile salts**	1.5 g
**Sodium chloride**	5.0 g
**Neutral red**	0.03 g
**Crystal Violet**	0.001 g
**Agar**	13.5 g
**Distilled water**	Add to make 1 Liter

**Table 3:** Composition of Perfringens agar base.

Ingredients	Amount (g)
**Tryptose**	15.0
**Soya peptone**	5.0
**Yeast extract**	5.0
**Sodium metabisulphite**	1.0
**Ferric ammonium citrate**	1.0
**Agar**	19.0
**Distilled water**	Add to make 1 Liter

*Lactobacillus* count was done using Rogosa agar (Table 4) plates and cultured by dilutions from 10^−1^ to 10^−7^, then incubated at 37°C in 5% CO_2_. All whitish colonies that appeared after 48 h of incubation were counted [23].

**Table 4:** Components of Rogosa agar.

Ingredients	Amount (g)
**Tryptone**	10.0
**Yeast extract**	5.0
**Glucose**	20.0
**Sodium acetate, anhydrous**	17.0
**Ammonium citrate**	2.0
**Potassium dihydrogen phosphate**	6.0
**Magnesium sulfate**	0.575
**Manganese sulfate**	0.120
**Ferrous sulfate**	0.034
**Bacteriological agar**	20
**Tween 80**	1 ml
**Distilled water**	Up to 1 L

### Hemagglutination inhibition (HI) assay

Antibody titers for Newcastle disease vaccine (NDV) were determined in each group (*n* = 10) at 21 and 35 d of age, with the HI test as recommended by Takatsy [24] and Brugh [25].

### Biochemical analysis

The collected sera were analyzed for total protein, albumin, alanine aminotransferase (ALT, EC 2.6.1.2), aspartate aminotransferase (AST, EC 2.6.1.1), triacylglycerol (TAG), total cholesterol, creatinine, and uric acid levels following the instructions enclosed in the manufactured kits produced by Biodiagnostic Company, Giza, Egypt. Serum globulin levels were calculated by subtraction of the albumin value from the total protein value of the same sample [26].

### RNA extraction and reverse transcription-polymerase chain reaction (RT-PCR)

RNA extraction from intestinal samples (*n* = 10) of all groups was done using Siam RNeasy Mini kit (Qiagen, GmbH, Hilden, Germany,) whereby 30 mg of the sample was added to 600 μl guanidine-thiocyanate-containing lysis buffer (RLT) containing 10 μl β-mercaptoethanol per 1 ml. For homogenization of samples, tubes were placed into the adaptor sets, which are fixed into the clamps of the Qiagen tissueLyser. Disruption was performed in a 2 min high-speed (30 Hz) shaking step. One volume of 70% ethanol was added to the cleared lysate, and the steps were completed per the purification of total RNA from animal tissues protocol of the QIAamp RNeasy Mini kit (Qiagen). Primers used were supplied from Metabion (Steinkirchen, Germany) for *β-actin* [27], Cu, Zn-superoxide dismutase (*SOD1*) and glutathione peroxidase (*GSH-Px*) [28], and *28S rRNA*, interferon-gamma (*IFN-γ*), interleukin-10 (*IL-10*), and interleukin-18 (*IL-18*) [29] as listed in Tables 5 and 6.

**Table 5:** Primers sequences, target genes, amplicon sizes, and cycling conditions for SYBR green RT-PCR.

Target genes	Primers sequences (5'→3')	Reverse transcription	Primary denaturation	Amplification (40 cycles)			Dissociation curve (1 cycle)		
				Secondary denaturation	Annealing (Optics on)	Extension	Secondary denaturation	Annealing	Final denaturation
***β-actin***	F: TGCTGTGTTCCCATCTATCG	50°C 30 min	94°C 5 min	94°C 15 sec	51°C 30 sec	72°C 30 sec	94°C 1 min	55°C 1 min	94°C 1 min
	R: TTGGTGACAATACCGTGTTCA								
***SOD1***	F: AGGGGGTCATCCACTTCC				60°C 30 sec			60°C 1 min	
	R: CCCATTTGTGTTGTCTCCAA								
***GSH-Px***	F: TTGTAAACATCAGGGGCAAA								
	R: ATGGGCCAAGATCTTTCTGTAA								

**Table 6:** Primers sequences, target genes, amplicon sizes and cycling conditions for Taqman RT-PCR.

Target genes	Primers and probes sequences (5'→3')	Reverse transcription	Primary denaturation	Amplification (40 cycles)	
				Secondary denaturation	Annealing and extension (Optics on)
***28S rRNA***	F: GGCGAAGCCAGAGGAAACT	50°C/30 min	94°C/5 min	94°C/15 sec	60°C/1 min
	R: GACGACCGATTTGCACGTC				
	(FAM) AGGACCGCTACGGACCTCCACCA (TAMRA)				
***IFN-γ***	F: AAACAACCTTCCTGATGGCGT				
	R: CTGGATTCTCAAGTCGTTCATCG				
	(FAM) TGAAAGATATCATGGACCTGGCCAAGCTC (TAMRA)				
***IL-10***	F: CATGCTGCTGGGCCTGAA				
	R: CGTCTCCTTGATCTGCTTGATG				
	(FAM) CGACGATGCGGCGCTGTCA (TAMRA)				
***IL-18***	F: GCCCTCCTCCTGGTTTCAG				
	R: TGGCACCGCAGCTCATT				
	(FAM) TCTTTACCAGCGTCCTACCTTGCGACA (TAMRA)				

SYBR Green RT-PCR primers were used in a 25 μl reaction containing 12.5 μl of the 2X QuantiTect SYBR Green PCR Master Mix (Qiagen), 0.25 μl of RevertAid Reverse Transcriptase (200 U/μl) (Thermo Fisher Scientific, Deutschland, Germany), 0.5 μl of each primer of 20 pmol/l concentration (Table 5), 8.25 μl of water, and 3 μl of RNA template. The reaction was performed in a Stratagene MX3005P RT-PCR machine.

Taqman RT-PCR PCR amplifications were conducted in a final volume of 25 μl containing 3 μl of RNA template, 12.5 μl of 2X QuantiTect Probe RT-PCR Master Mix, 8.125 μl PCR grade water, 0.5 μl of each primer of 20 pmol concentration (Table 6) and 0.125 μl of each probe (30 pmol/l) and 0.25 μl of QuantiTect RT Mix. The reaction was performed in a Stratagene MX3005P RT-PCR machine.

The Stratagene MX3005P software determined amplification curves and CT values. To estimate the variation of gene expression on the RNA of the different samples, the CT of each sample was compared with that of the positive control group per the "ΔΔCt” method by using the following ratio: (2^-ΔΔct^) [30].

### Histology

Specimens (*n* = 10) were collected from jejunum of each group and rapidly fixed in 10% neutral buffered formalin solution for at least 24 h. The fixed samples were processed with the conventional paraffin embedding technique. Histological sections of 3 μm were prepared from the paraffin blocks of samples. These sections were stained with Hematoxylin and Eosin (H&E) technique [31]. The measurements of the villi lengths and crypts depth in control and lysozyme-supplemented groups were made using ImageJ software, version 1.48 (National Institutes of Health, Bethesda, MD, USA; http://rsb.info.nih.gov/ij/).

### Statistical analyses

Statistical measurements were handled with the SPSS programming tool (IBM SPSS. 20^®^, Coppell, TX, USA) using One-way ANOVA followed by Duncan’s multiple range tests. Data obtained from the intestinal bacterial counts, HI assay, and RT-PCR assays were analyzed with One-way ANOVA, Tukey’s *post hoc* multiple range tests with GraphPad Prism 5 (San Diego, CA, USA). All declarations of significance depended on *P* < 0.05.

## Results

### Growth performance and survival

Regarding the amount of lysozyme consumed by birds in lysozyme-supplemented groups, the individual bird consumed 0.557 mg of lysozyme per day in LYZ70, 0.711 mg in LYZ90, and 0.966 mg in LYZ120.

At the start of the experiment, there was no significant (*P* > 0.05) differences in the live body weight between different experimental groups (Table 7). Throughout the experimental period, compared to control group, lysozyme supplementation improved growth by 2.92, 5.39, and 3.14% in LYZ70, LYZ90, and LYZ120 groups, respectively, while the feed intake was unaffected (*P* > 0.05). The body weight gain and feed conversion ratios (FCR) were significantly (*P* ˂ 0.05) improved in LYZ90 group only. Similarly, at the end of the experiment, protein efficiency ratio (PER) was unaffected (*P* > 0.05), but the European production efficiency factor (EPEF) and European broiler index (EBI) were significantly (*P* ˂ 0.05) improved in LYZ90, LYZ70, and LYZ120 with percentages of 17, 12.76, and 12.46, respectively (S1 Data). Viability percentages were 100% in all lysozyme supplemented groups compared to 93.33% value in control. Mortality was nil in lysozyme-supplemented groups as against 6.67 percent mortality in control birds.

**Table 7:** Effect of lysozyme dietary supplementation on growth performance of broiler chickens.

Items (±SE) ^‡^	Periods	Control	LYZ70	LYZ90	LYZ120
^**1**^**BW (g)**	0 d	41.13 ± 0.76	40.75 ± 0.88	41.83 ± 0.97	41.10 ± 0.87
^**2**^**BWG (g/bird/week)**	0–21 d	733.20 ± 13.04	777.75 ± 15.86	771.83 ±14.17	773.40 ± 15.62
	0–35 d	1730 ± 32.87 ^b^	1783.25 ± 35.72 ^ab^	1829.93 ± 32.07 ^a^	1787.07 ± 28.92 ^ab^
^**3**^**VFI (g/bird/week)**	0–21 d	1023.39 ± 2.45	1024.39 ± 1.61	1016.76 ± 8.20	1031.56 ± 2.78
	0–35 d	2808.55 ± 10.99	2795.89 ± 25.92	2763.08 ± 32.73	2816.56 ± 15.66
^**4**^**FCR**	0–21 d	1.41 ± 0.03	1.33 ± 0.03	1.33 ± 0.02	1.35 ± 0.03
	0–35 d	1.64 ± 0.04 ^a^	1.58 ± 0.03 ^ab^	1.54 ± 0.03 ^b^	1.59 ± 0.03 ^ab^
^**5**^**PER**	0–21 d	3.12 ± 0.06	3.30 ± 0.07	3.30 ± 0.05	3.26 ± 0.07
	0–35 d	2.97 ± 0.06	3.07 ± 0.06	3.15 ± 0.05	3.05 ± 0.05
^**6**^**EPEF**	0–21 d	261.39 ± 1.99 ^b^	293.06 ± 1.26 ^a^	291.60 ± 8.61 ^a^	283.16 ± 4.86 ^a^
	0–35 d	288.05 ± 19.78 ^b^	330.18 ± 10.21 ^a^	347.58 ± 4.19 ^a^	329.01 ± 1.97 ^a^
^**7**^**EBI**	0–21 d	256.09 ± 6.95 ^b^	282.18 ± 0.89 ^a^	303.59 ± 18.59 ^a^	308.10 ± 5.72 ^a^
	0–35 d	281.63 ± 19.39 ^b^	322.83 ± 10.20 ^a^	339.87 ± 4.02 ^a^	321.63 ± 2.14 ^a^

Statistical analysis was done ANOVA followed by Duncan’s multiple range tests

‡ Values are given as mean ±Standard error (SE) of triplicates, each replicate contained 10 birds.

Any two means for a performance parameter bearing different superscript letters in a row are significantly (*P* < 0.05) different from each other. Therefore, ‘‘a” and ‘‘b” letters in the same row are significantly different, while ‘‘ab” and ‘‘b” are non-significantly different.

Abbreviations

^1^ Body weight

^2^Body weight gain

^3^Voluntary feed intake

^4^Feed conversion ratio

^5^Protein efficiency ratio.

^6^European production efficiency factor = [(viability % × body weight Kg / age (d) × FCR)] ×100

^7^European broiler index = [viability % × average daily gain (g/chick/day) / FCR (kg feed/kg gain)] ×10

### Intestinal bacterial counts

**Fig 1:**
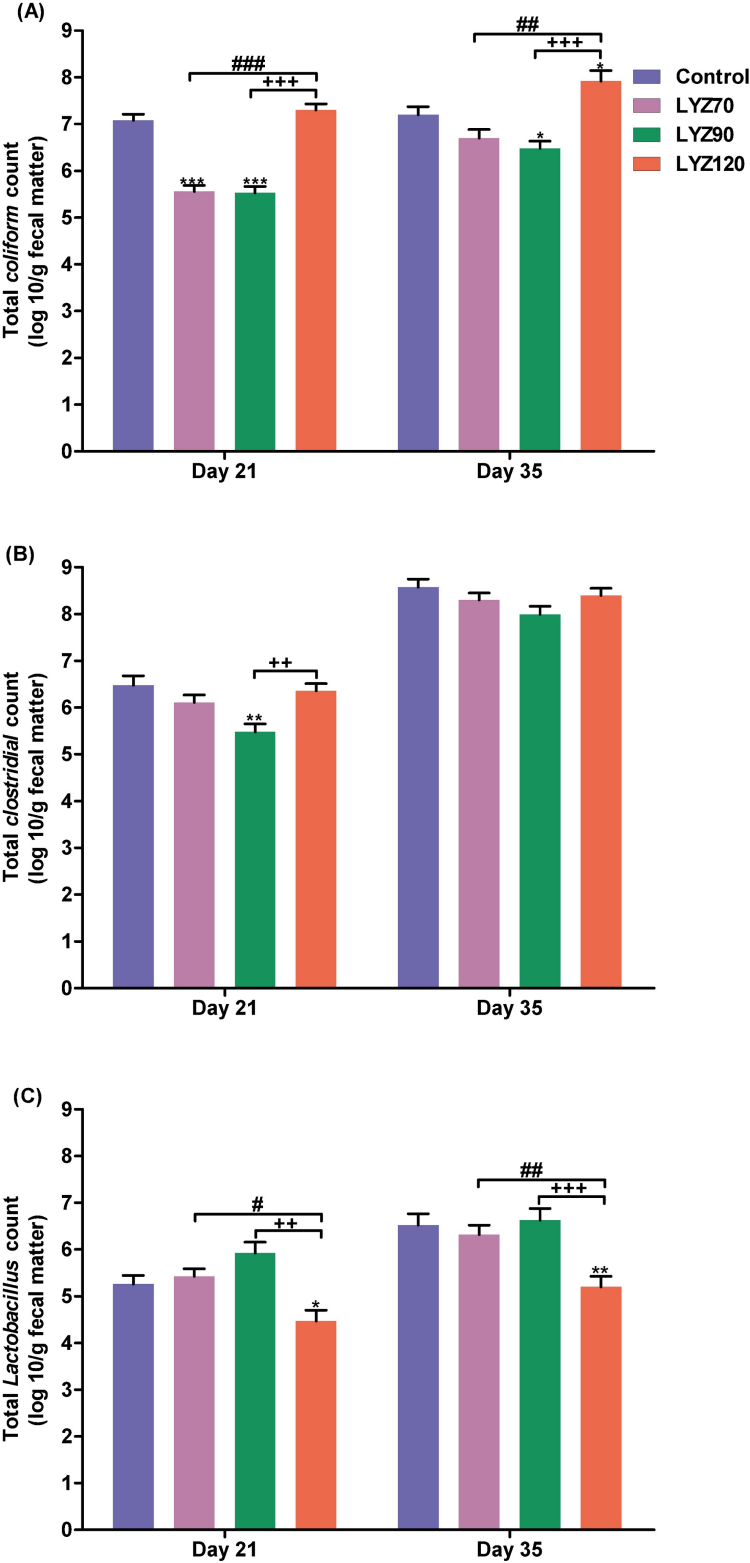
Total *Coliform*, *Clostridial*, and *lactobacillus* counts in fecal samples. *P < 0.05, **P < 0.01, and ***P < 0.001 vs. control, ^++^*P* < 0.01 and ^+++^*P* < 0.001 vs. LYZ90, and ^#^*P* < 0.05, ^##^*P* < 0.01, and ^###^*P* < 0.001 vs. LYZ120.

Regarding the intestinal microbiota, the total *Coliform* count in LYZ70 and LYZ90 groups were significantly (*P* ˂ 0.001) lower than control or LYZ120 at 21 d, while at 35 d, the lowest group was LYZ90 (S1 Data). For total *Clostridial* count, LYZ90 gave the lowest number at 21 and 35 d of age compared with other groups, while LYZ90 resulted in the highest *Lactobacillus* count at 21 and 35 d of age compared with other groups (Fig 1).

### Hemagglutination inhibition (HI) test

**Fig 2:**
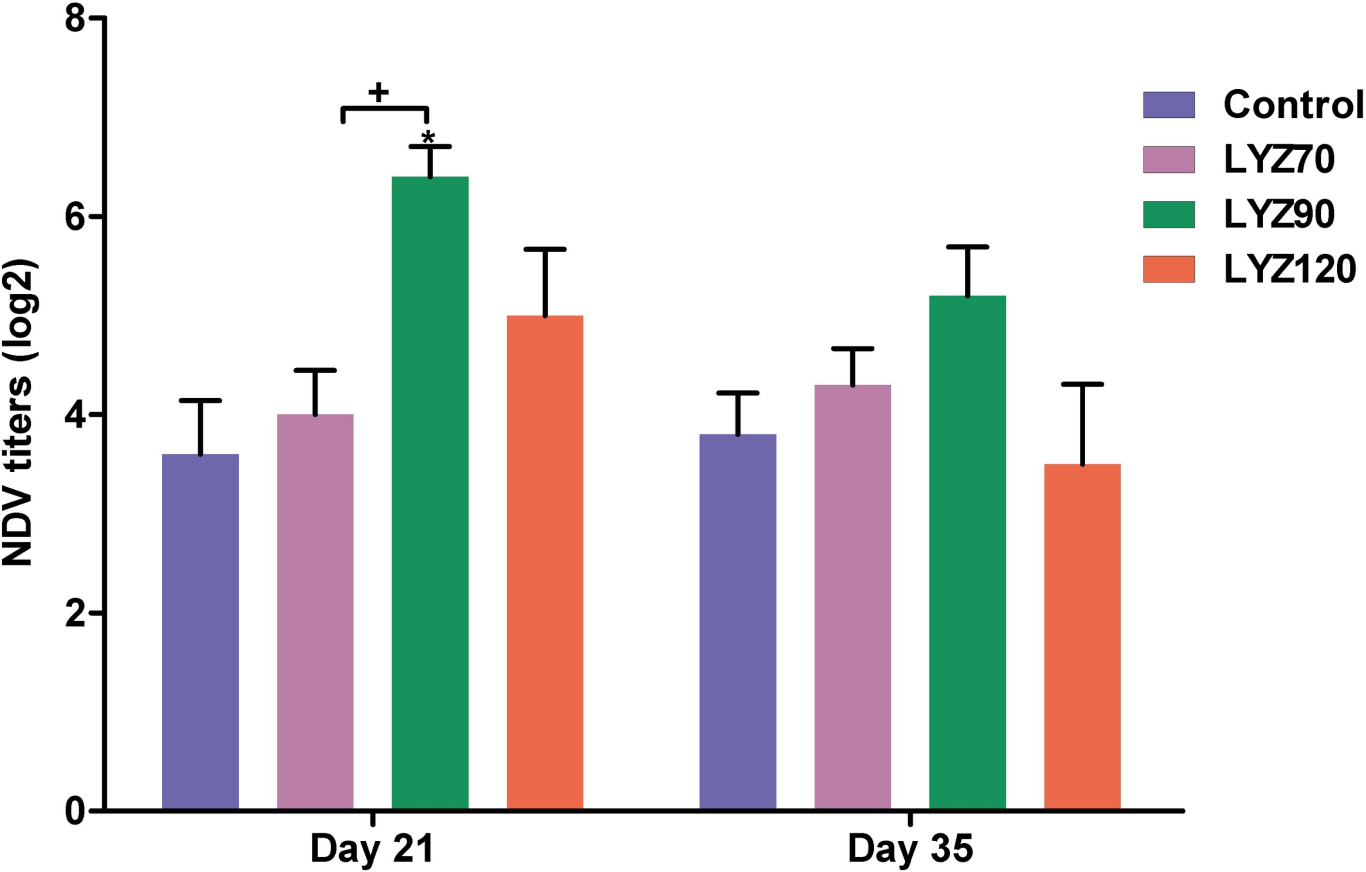
Hemagglutination inhibition (HI) titer for Newcastle disease vaccine (NDV). *P < 0.05 vs. control and ^+^*P* < 0.05 vs. LYZ90. Statistical analysis was done by One-way ANOVA, Tukey’s *post hoc* test multiple comparisons. Error bars represent SE.

Data illustrated in Fig 2 shows increment in serum NDV antibody titers in lysozyme-supplemented groups. The antibody titers were significantly increased (*P* < 0.05) in LYZ90 when compared to control group at 21 d and non-significantly increased (*P* > 0.05) at 35 d. Also, the titers were increased significantly (*P* < 0.05) in LYZ90 when compared with LYZ70 at 21 d (S1 Data).

### Biochemical analysis

Serum globulin values were significantly increased (*P* < 0.05) in lysozyme-supplemented groups in a dose-related manner at 21 and 35 d in comparison to control (Table 8). For liver function (ALT and AST), kidney function (creatinine and uric acid), and lipid profile (TAG and total cholesterol) biomarker values, the current study found no significant differences (*P* > 0.05) in these parameters in lysozyme-supplemented birds comparable to the control birds during the experimental period (S1 Data).

**Table 8:** Effect of lysozyme dietary supplementation on serum total protein (g/dl), albumin (g/dl), globulin (g/dl), ALT (U/L), AST (U/L), TAG (mg/dl), total cholesterol (mg/dl), creatinine (mg/dl), and uric acid (mg/dl) in broiler chickens.

	Items (±SE) ^‡^	Control	LYZ70	LYZ90	LYZ120
**21 d**	**Total protein**	4.46 ± 0.17 ^b^	5.13 ± 0.24 ^a^	4.40 ± 0.06 ^b^	4.98 ± 0.08 ^a^
	**Albumin**	3.57 ± 0.18 ^a^	3.62 ± 0.08 ^a^	3.14 ± 0.08 ^b^	3.27 ± 0.06 ^ab^
	**Globulin**	0.89 ± 0.22 ^b^	1.51 ± 0.19 ^a^	1.26 ± 0.10 ^ab^	1.71 ± 0.11 ^a^
	^**1**^**ALT**	19.10 ± 3.36	18.52 ± 0.39	22.50 ± 0.95	22.26 ± 0.29
	^**2**^**AST**	200.78 ± 3.41	209 ± 1.66	206.33 ± 0.93	208.93 ± 6.80
	^**3**^**TAG**	135.79 ± 2.02	137.23 ± 5.45	132.84 ± 1.35	136.22 ± 0.67
	**Total cholesterol**	106.96 ± 10.48	113.03 ± 9.63	88.83 ± 4.01	118.34 ± 17.82
	**Creatinine**	0.33 ± 0.03	0.33 ± 0.03	0.36 ± 0.04	0.34 ± 0.01
	**Uric acid**	9.29 ± 1.50 ^a^	9.28 ± 0.85 ^a^	6.74 ± 0.69 ^ab^	5.62 ± 0.34 ^b^
**35 d**	**Total protein**	5.22 ± 0.16 ^b^	5.70 ± 0.18 ^b^	5.39 ± 0.23 ^b^	6.77 ± 0.93 ^a^
	**Albumin**	3.61 ± 0.17 ^a^	3.73 ± 0.08 ^a^	3.29 ± 0.07 ^b^	3.82 ± 0.04 ^a^
	**Globulin**	1.61 ± 0.30 ^b^	1.97 ± 0.14 ^ab^	2.10 ± 0.17 ^ab^	2.69 ± 0.63 ^a^
	^**1**^**ALT**	17.27 ± 2.29	16.71 ± 3.33	13.90 ± 1.75	16.32 ± 0.83
	^**2**^**AST**	207.33 ± 3.15	208.77 ± 2.44	204.18 ± 2.31	206.66 ± 3.54
	^**3**^**TAG**	133.56 ± 4.43	130.41 ± 2.40	130.01 ± 3.03	139.32 ± 1.38
	**Total cholesterol**	108.22 ± 13.66	129.93 ± 8.73	113.58 ± 7.88	145.41 ± 17.84
	**Creatinine**	0.51 ± 0.05	0.48 ± 0.02	0.46 ± 0.04	0.49 ± 0.05
	**Uric acid**	8.37 ± 1.44	6.60 ± 0.52	6.12 ± 0.47	6.35 ± 0.97

‡ SEMs bearing different superscript letters are significantly (*P* < 0.05) different from the other values within the same column. Therefore, ‘‘a” and ‘‘b” letters in the same row are significantly different, while ‘‘ab” and ‘‘b” are non-significantly different.

Statistical analysis was done by Duncan’s multiple range tests.

Biochemical analysis was done in triplicate (*n* = 10).

Abbreviations

^1^Alanine aminotransferase

^2^Aspartate aminotransferase

^3^Triacylglycerol

### Expression of antioxidant enzymes- and immune-related genes

**Fig 3:**
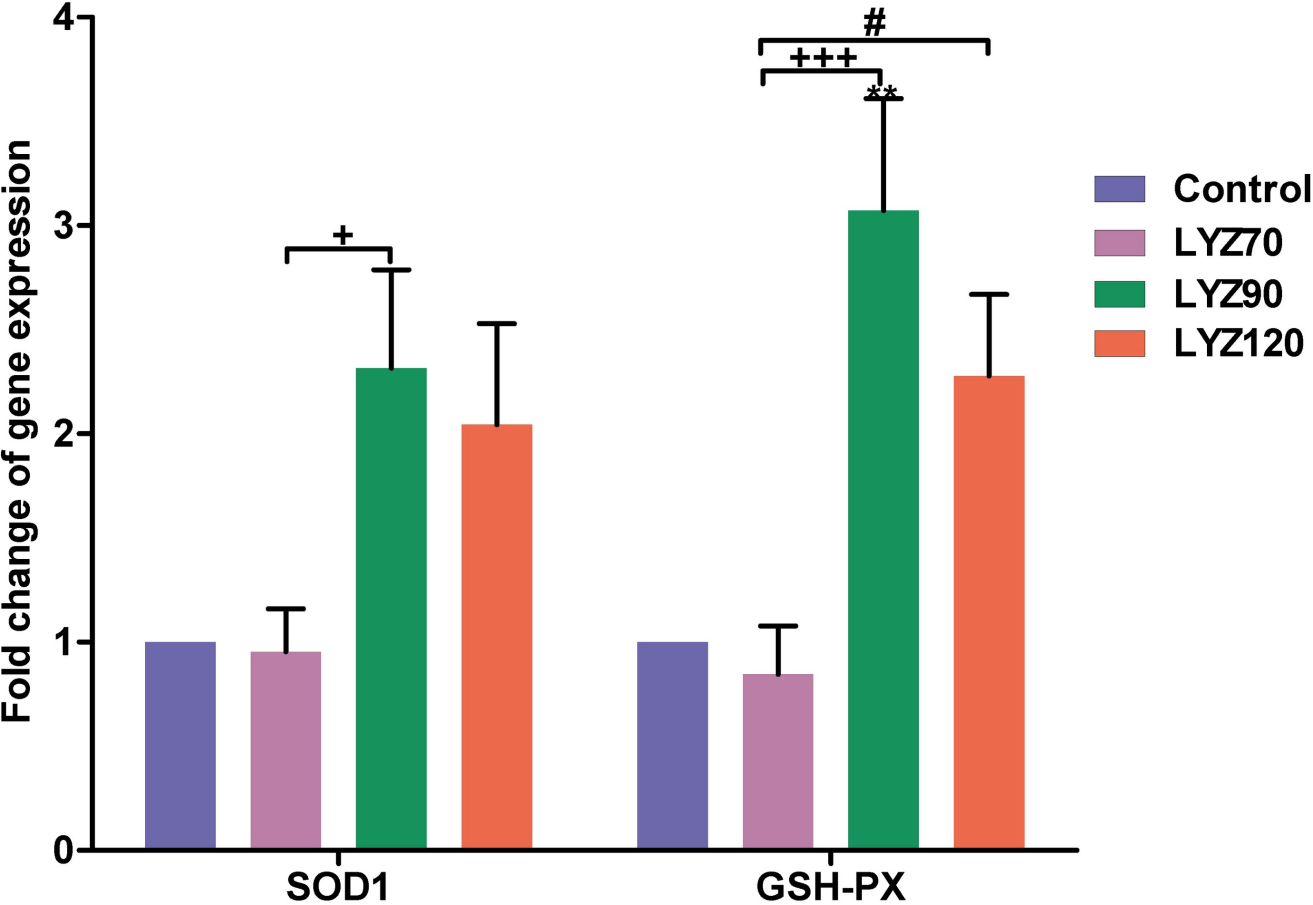
Reverse transcription polymerase chain reaction (RT-PCR) validation of selected genes differentially expressed due to lysozyme (LYZ). Selected genes were *SOD1* = Cu, Zn-superoxide dismutase and *GSH-Px* = glutathione peroxidase. **P < 0.01 vs. control, ^+^*P* < 0.05 and ^+++^*P* < 0.001 vs. LYZ90, and ^#^*P* < 0.05 vs. LYZ120. Statistical analysis was done by One-way ANOVA, Tukey’s *post hoc* test multiple comparisons. Error bars represent SE.

In the ileum samples, mRNA expression of the *SOD1* and *GSH-Px* were increased in LYZ90 and LYZ120 (Fig 3). The expression fold of *SOD1* was significantly increased (*P* < 0.05) in LYZ90 compared to LYZ70. *GSH-Px* gene expression was also significantly increased in LYZ90 when compared with control (*P* < 0.01) and LYZ70 (*P* < 0.001). Birds in LYZ120 had a significant change in mRNA expression fold (*P* < 0.05) compared with LYZ70 (S1 Data).

**Fig 4:**
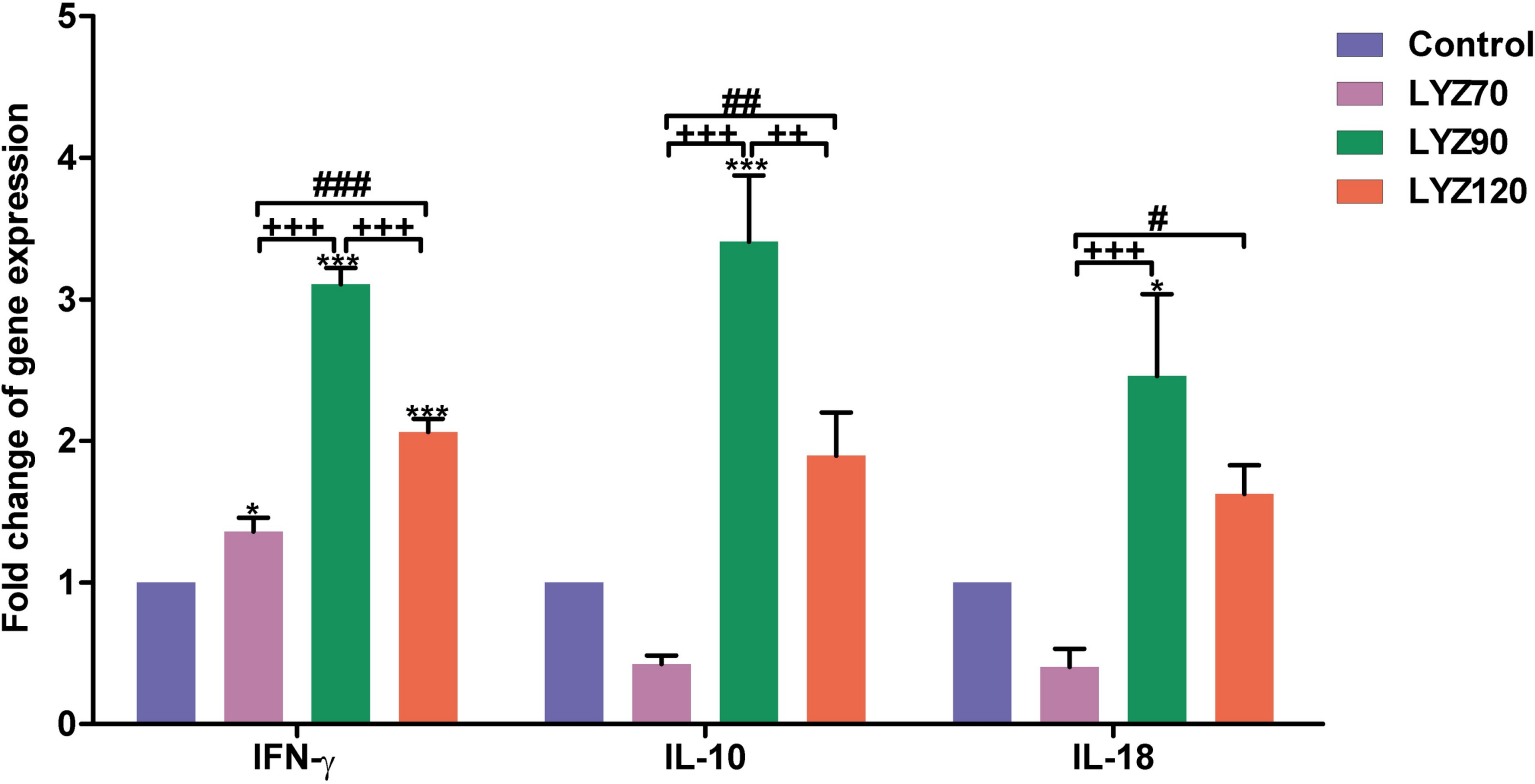
Reverse transcription polymerase chain reaction (RT-PCR) validation of selected genes differentially expressed due to lysozyme (LYZ). Selected genes were *IFN-γ* = interferon-gamma; *IL-10* = interleukin-10, and *IL-18* = interleukin-18. *P < 0.05 and ***P < 0.001 vs. control, ^++^*P* < 0.01 and ^+++^*P* < 0.001 vs. LYZ90, and ^#^*P* < 0.05, ^##^*P* < 0.01, and ^###^*P* < 0.001 vs. LYZ120. Statistical analysis was done by One-way ANOVA, Tukey’s *post hoc* test multiple comparisons. Error bars represent SE.

The expression of *INF-γ*, *IL-10*, and *IL-18* mRNA in different groups is given in Fig 4. The expression folds of *INF-γ* were significantly increased (*P* < 0.05) in LYZ70 and significantly increased (*P* < 0.001) in LYZ90 and LYZ120 when compared to control. Also, LYZ90 values were significantly increased (*P* < 0.001) when compared with either LYZ70 or LYZ120. The LYZ120 group exhibited a significant increase (*P* < 0.001) in expression fold compared with LYZ70.

Results of the *IL-10* gene expression showed a significant increase in LYZ90 (*P* < 0.001) compared to control. Also, there was a significant increase in LYZ90 compared with either LYZ70 (*P* < 0.001) or LYZ120 (*P* < 0.01). *IL-10* were significantly increased in LYZ120 (*P* < 0.01) in comparison to LYZ70

Results of the *IL-18* gene expression, showed that lysozyme dietary supplementation significantly increased the *IL-18* mRNA content in LYZ90 compared to control (*P* < 0.05) and LYZ70 (*P* < 0.001).

### Histology

**Fig 5:**
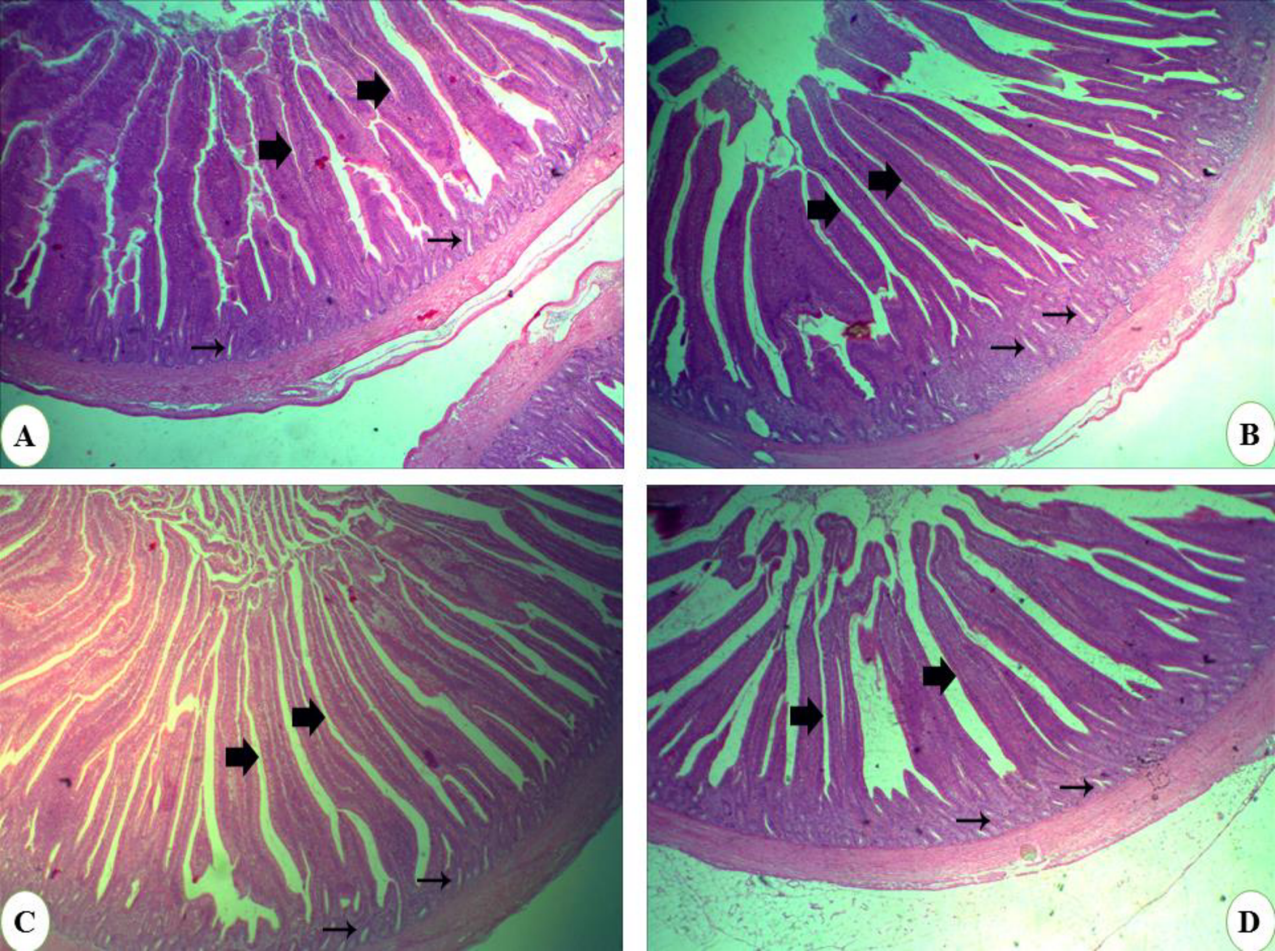
Histological examination of jejunum samples stained with H&E. (A) control (×40), (B) LYZ70 (×40), (C) LYZ90 (×100), and (D) LYZ120 (×40). Large arrows point to intestinal villi. Small arrows point to intestinal crypts.

Histological quantitation of exogenous lysozyme dietary supplementation was done by measuring villi length and crypt depth (Table 9 and Fig 5). The length of intestinal villi was significantly increased in response to lysozyme supplementation in LYZ70 and significantly increased in LYZ90. The crypt depth measures were significantly increased in lysozyme-supplemented groups (S1 Data).

**Table 9:** Effect of lysozyme dietary supplementation on villi length (μm) and crypt depth (μm) in broiler chickens (3 replicates per group).

	Control (±SE)	LYZ70 (±SE) ^‡^	LYZ90 (±SE) ^‡^	LYZ120 (±SE) ^‡^
Villi length	6.02 ± 0.14 ^c^	7.30 ± 0.08 ^b^	10.30 ± 0.08 ^a^	6.24 ± 0.23 ^c^
Crypt depth	0.86 ± 0.03 ^d^	1.32 ± 0.03 ^b^	1.69 ± 0.05 ^a^	1.16 ± 0.03 ^c^

‡ SEMs bearing different superscript letters are significantly (*P* < 0.05) different from the other values within the same column. Therefore, ‘‘a”, ‘‘b”, ‘‘c”, and ‘‘d” in the same row are significantly different. Statistical analysis was done by Duncan’s multiple range tests.

Villi length and crypt depth analysis were done in triplicate (*n* = 10).

## Discussion

### Growth performance

The presence of healthy and functional gut is imperative to maintain high feed efficiency and excellent production index. Dietary supplementation of broiler chickens with lysozyme increased the BWG and improved the FCR, especially in the LYZ90 group. Similarly, Liu et al. [11] recorded that lysozyme significantly improved the FCR and tended to increase weight gain in broiler chickens. Also, piglets that were fed a diet containing 90 mg lysozyme/kg had growth performance greater than antibiotic-treated, 120 mg lysozyme/kg, and control groups [32]. The effect of lysozyme on growth performance may be due to the enhanced gut antioxidant and immune genes besides the significant increase in intestinal villi, which concomitantly increase the intestinal surface area for nutrients absorption [33].

Higher doses of lysozyme, as evidenced by the current study, induced the poorest growth performance. The same results were reported in piglet [32]. Lysozyme supplementation in higher doses might suppress the growth of *Lactobacillus*, which can improve the growth performance of broiler chickens [34] through stimulation of the enterocyte growth with an increase in intestinal villi and crypts favoring the absorption of digested nutrient [35].

### Biochemical analysis

No adverse effects for lysozyme on liver and kidneys function tests in broiler chickens were found, which indicates the safety of lysozyme supplementation. Moreover, lysozyme had no effect on the serum TAG and total cholesterol in comparison to control indicating no direct correlation between lysozyme and lipid profiles. But we determined the total cholesterol and TAG levels because they are synthesized in the liver and are considered as markers for hepatic synthesis efficiency. The results are in accordance with the general requirements of animal feed additives in that they must be nutritive, health improving, and harmless to the animal and consequently, produce safe products for human [36].

### Intestinal bacterial count and immunity-enhancement

*Lactobacillus* is a Gram-positive beneficial bacterium for animals found in the crop and intestine. The addition of LYZ90 increased the total *Lactobacillus* count compared to other groups, especially LYZ120, which markedly decreased. This decrease in *Lactobacillus* count in LYZ120 was accompanied by a downregulation in *IFN-γ*, which is a mediator of mucus that has antimicrobial roles, a physical barrier, as well as being composed of mucin glycoproteins that are directly toxic to many harmful bacteria. On the contrary, LYZ90 decreased both *Coliforms* and *Clostridial* counts as well as increased *Lactobacillus* counts because it induced a high mucous secretion due to the noticeable enhancement of intestinal *IFN-γ* gene. Jiménez-Saiz et al. [37] and Mine et al. [38] stated that oral lysozyme could be hydrolyzed in the duodenum and be accompanied by the production of antimicrobial peptides that could play critical roles in innate immunity. Also, the beneficial effect of lysozyme on gut morphology could be attributable to changing the intestinal microflora and could result in decreased coliform count [39,40] leading to maturation of the intestinal tract [41] that promote nutrient digestion and gastrointestinal absorption, which finally led to growth enhancement [32]. In addition, the villi length and crypt depth were elongated in lysozyme-supplemented groups with an obvious improvement with LYZ90. The increased villus length increased the surface area facing the digested nutrient that facilitates absorption [42].

Intestinal microbiota and antioxidant enzymes modulate the gut immunity that ultimately is reflected in serum globulin levels and disease resistance as measured through HI test and survival percentages. The increment in the antibody titers against NDV vaccination in the lysozyme-supplemented groups indicates that the enhancement in the bird immunity because of the antioxidant and immune status improvements. These results corroborate those of Fritz et al. [43] and Siwicki et al. [44]. Similar improvement in the serum globulin values in a dose-dependent manner in lysozyme fed birds supports its immune stimulatory role.

In the present study, exogenous lysozyme induced overexpression of intestinal *SOD1* and *GSH-Px*, leading to a marked increment in the intestinal detoxification status against various xenobiotics. An *in vivo* study on the effect of dietary lysozyme on the antioxidant potential of juvenile gibel carp recorded a significant increase in the serum SOD and a significant decrease in malondialdehyde of the mid-intestine along with increased goblet cells in mid-intestine and microvilli height [45].

Data obtained in this study revealed significant increases in *IFN-γ*, *IL-10*, and *IL-18* gene expressions of ileal samples in response to exogenous lysozyme supplementation in broiler chickens with a noticeable enhancement in the LYZ90. In the same manner, exogenous lysozyme augmented the immune function in adult pigeons [46] and dimerized lysozyme stimulated the cellular and humoral defense mechanisms in rainbow trout [44].

## Conclusion

The results of this study showed that the exogenous lysozyme supplementation to broiler chickens improved the bird’s growth performance and enhanced intestinal health through positive modulation of gut microbiota and expression of genes involved in gut antioxidant status and nonspecific immunity. A dose rate of 90 g lysozyme 10%^®^ per ton of basal diet is recommended for broiler chickens.

## Supporting information

S1 Data Raw data of growth performance, bacterial counts, hemagglutination inhibition test, biochemical analysis, RT-PCR, and histological analysis. (XLSX)

S1 Table Arrive guideline checklist. (DOCX)
