# Supplementary material for: Exogenous dietary lysozyme improves the growth performance and gut microbiota in broiler chickens targeting the antioxidant and non-specific immunity mRNA expression
Source: PLoS One. 2017 Oct 23;12(10):e0185153. doi: 10.1371/journal.pone.0185153 (PMC5653193; doi:10.1371/journal.pone.0185153)

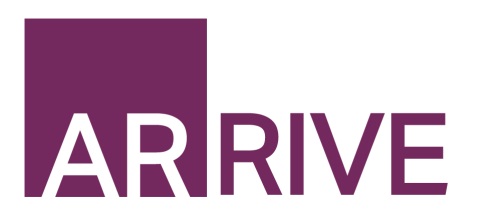


The ARRIVE Guidelines Checklist

Animal Research: Reporting In Vivo Experiments

Carol Kilkenny^1^, William J Browne^2^, Innes C Cuthill^3^, Michael Emerson^4^ and Douglas G Altman^5^

*^1^The National Centre for the Replacement, Refinement and Reduction of Animals in Research, London, UK, ^2^School of Veterinary Science, University of Bristol, Bristol, UK, ^3^School of Biological Sciences, University of Bristol, Bristol, UK, ^4^National Heart and Lung Institute, Imperial College London, UK, ^5^Centre for Statistics in Medicine, University of Oxford, Oxford, UK.*

|  | | ITEM RECOMMENDATION | Section/ Paragraph |  |
| --- | --- | --- | --- | --- |
| 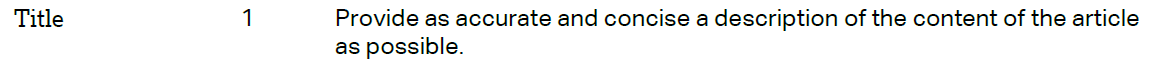 | | Exogenous Dietary Lysozyme Improves the Growth Performance and Gut Microbiota in Broiler Chickens Targeting the Antioxidant and Non-specific Immunity mRNA Expression |  |  |
| 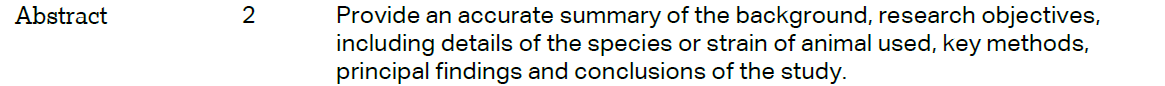 | | Healthy gut and proper immunity in broiler chickens is the key for improving growth performance. Therefore, the current study was conducted to investigate the effect of the dietary supplementation of exogenous lysozyme on the growth performance and immunity of broiler chickens. Experimentally, 120 one-day-old Ross 308 chicks were randomly allocated into 4 groups and 3 replicates per group (30 birds/group). The chicks were fed on the starter (1^st^ - 21^st^ days) and grower (22^nd^ - 35^th^ days) diets supplemented with 0 (CON), 70 (LYZ70), 90 (LYZ90) and 120 (LYZ120) g/ton of lysozyme 10%^®^, respectively, for 5 weeks. The results revealed significant enhancement in growth performance traits and improvement in the gut environment by a significant decrease in fecal *Coliform* and *Clostridial* counts and a significant increase (*P* ˂ 0.05) in the beneficial *Lactobacillus* in the lysozyme-treated group, especially LYZ90. Moreover, the mRNA expressions of SOD1, GSH-Px, IFN-γ, IL-10, and IL-18 were up-regulated in response to lysozyme supplementation. In comparison to CON, the birds in LYZ90 have significant increase (*P < 0.01*) in the GSH-Px gene expression that enhance the antioxidant status of gut. Regarding the gut non-specific immunity, significant increases the expression folds of INF-γ (*P < 0.001*), IL-10 (*P < 0.001*), and IL-18 (*P < 0.05*) were recognized in LYZ90. Serum globulin levels were significantly elevated (*P* ˂ 0.05) in lysozyme-treated groups. Intestinal villi length and crypts depth were also enhanced (*P* ˂ 0.05) by lysozyme dietary supplementation. In conclusion, supplementation of broiler chickens with exogenous lysozyme, in particular with the concentration of 90 g of lysozyme 10%^®^ per ton of basal diet, improved the gut non-specific immunity and antioxidant status as monitored through enhancement of the broiler chickens’ growth performance and gut microbiota. |  |  |
| INTRODUCTION | |  |  |  |
| 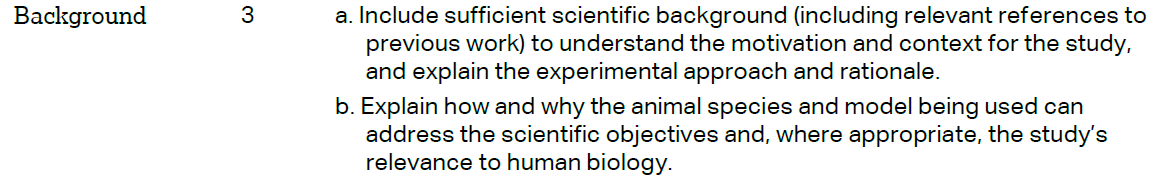 | | Lysozyme (EC 3.2.1.17) is a natural antimicrobial protein considered an important component of the innate immune system. It exerts bacteriolytic activity by hydrolyzing the β-1,4-glycosidic linkage between N-acetylmuramic acid and N-acetyl glucosamine of bacterial cell wall mainly against many Gram-positive bacteria (Ibrahim et al., 1994). Because of its abundance in egg white, lysozyme is commercially extracted from the eggs and has been applied as a natural food preservative and a therapeutic drug for humans. Also, the in vivo intraperitoneal administration of lysozyme decreases the pathology resulting from a Klebsiella pneumoniae in mice (Ivanovska et al., 1996). Thus, it provides protection against bacterial diseases. However, there are few reports concerning the exogenous lysozyme effect on poultry diseases. In vitro studies stated the antimicrobial activity of exogenous lysozyme against *Clostridium perfringens* type A in broiler chickens which associated with necrotic enteritis. They reported that lysozyme could control *C. perfringens* type A. Also, exogenous lysozyme reduced the number of *C. perfringens* in the ileum of broiler chickens and prevented intestinal lesions when they were administered by gavage with *C. perfringens* (Liu et al., 2010). The changes in microbiota may reduce the adverse effect of anti-nutritional factors in feeds modulate microbiota, immune function, improve the intestinal morphology and decrease the gut oxidative stress (Lallès, 2016).  We used the broiler chickens to determine the effect of exogenous lysozyme on the production of healthy birds with no antibiotic supplementation suitable for human consumption |  |  |
| 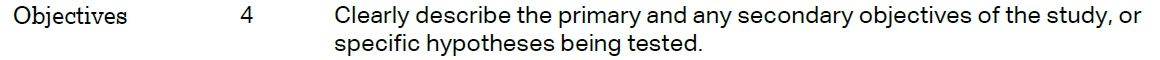 | | There are limited results focused on the effect of different levels of exogenous lysozyme supplementation on broiler’s performance and gut health through gut integrity, morphology, and intestinal microbiota in addition to its impact on immune responses.  Produce poultry meat product with no antibiotic for human consumption. |  |  |
| METHODS | |  |  |  |
| 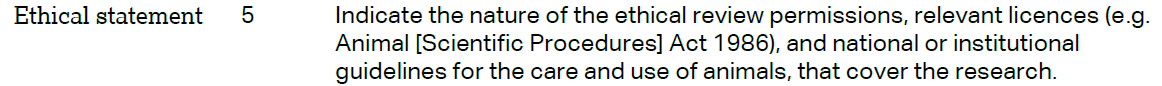 | | This study was carried out in strict accordance with the recommendations of the Committee on the Ethics of Animal Experiments of Damanhour University, Egypt. All procedures and experiments complied with the guidelines and were approved by the Local Ethic Commission of the Animal Experiments of Damanhour University with respect to animal experimentation and care of animals under study, and all efforts were made to minimize suffering. |  |  |
| 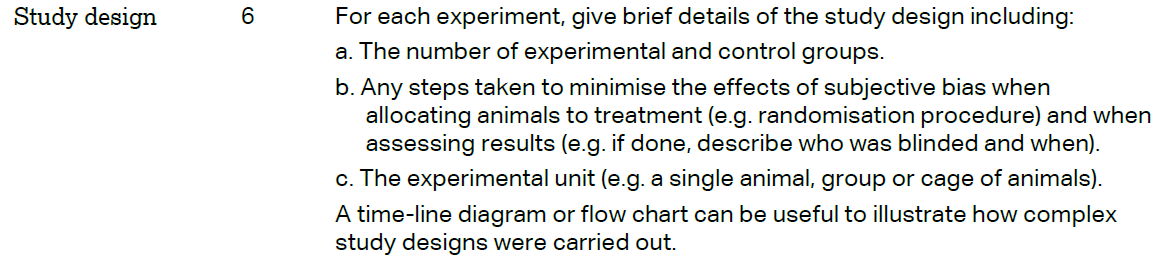 | | In total, 120 one-day-old Ross 308 chicks were obtained from a commercial hatchery and randomly distributed into four groups of mixed sexes (30 birds per group). Each group was subdivided into three replicates per group and reared on wire-floored cages of the same dimensions with the same number of nipple drinkers, feed hoppers, and received an experimental diet for five consecutive weeks. The birds were freely allowed to consume feed and water ad libitum and kept under daily observations. The incubation temperature of 32°C was gradually decreased until reaching 26°C by the 3rd week of age and the chicks were exposed to a 23 h light.  The chicks were allocated into a control group 1 (CON, fed on a commercial basal diet), group 2 (LYZ70), group 3 (LYZ90), and group 4 (LYZ120) were fed on a commercial basal diet containing 70, 90, and 120g lysozyme 10%® (Nan Chang Lifeng Industry and Trading Co., Ltd., Jiangxi, China) per ton, respectively. Hence, the individual bird per day consumed 0.557 mg (group 2), 0.711 mg (group 3), and 0.966 mg (group 4). Regarding lysozyme 10%® each 1 kg contains 100 g of lysozyme, 50 g of glycine, 10 g of asparagic acid, 8 g of water and 832 g glucose.  The basal diets (corn-soybean based diet) of starter phase (1 - 21 days) and grower phase (22 - 35 days) met the recommendation of National Research Council Nutrient Requirements for broiler chickens [17]. The ingredients and nutrient composition of the basal diet were analyzed according to AOAC [18] and it is shown in Table 1. The metabolizable energy in the basal diet in (Kcal/kg diet) was calculated following the NRC. Interestingly, the birds of all groups did not receive any antibiotic supplementation along the experimental period. |  |  |
| 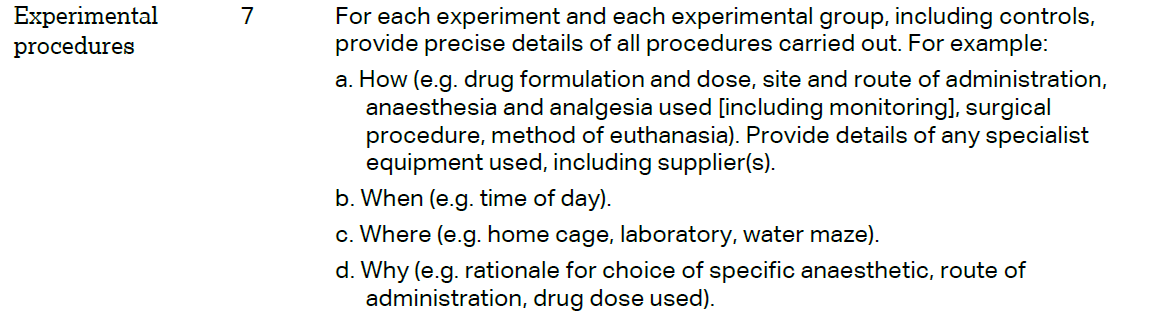 | | Blood samples (n=10) were collected from wing vein at the 21 and 35 days of age without anticoagulant for serum separation. Collected samples were centrifugated at 4000 rpm for 5 min at 4°C to obtain clear sera for HI test against NDV and biochemical analysis.  At the end of the experiment (35th day), five birds from each group were sacrificed under anesthesia with intravenous injection of sodium pentobarbital (50 mg/kg) and necropsied immediately. Samples of 1 cm were taken from ileum (5 cm from Meckel’s diverticulum) and washed immediately with physiological saline (0.9% NaCl). Each sample was kept in an Eppendorf tube and immersed instantly in the liquid nitrogen. Additionally, samples of 3 cm were obtained from jejunum (1 cm cut from the midpoint) were washed with saline and kept in 10% neutral buffered formalin for 24 h for histological examination. |  |  |
| 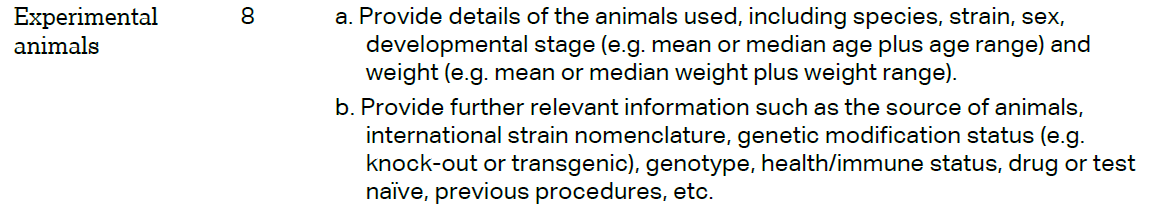 | | One-day-old Ross 308 chicks were reared on wire-floored cages.  Ross 308 (*Gallus gallus*) |  |  |

The ARRIVE guidelines. Originally published in *PLoS Biology*, June 2010^1^

| 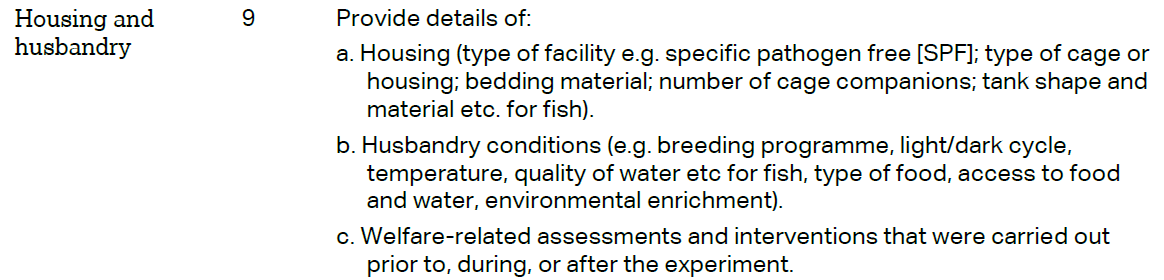 | The birds were reared on wire-floored cages of the same dimensions with the same number of nipple drinkers, feed hoppers, and received an experimental diet for five consecutive weeks. The birds were freely allowed to consume feed and water ad libitum and kept under daily observations. The incubation temperature of 32°C was gradually decreased until reaching 26°C by the 3rd week of age and the chicks were exposed to a 23 h light.  At the end of the experiment (35th day), five birds from each group were sacrificed under anesthesia with intravenous injection of sodium pentobarbital (50 mg/kg) and necropsied immediately. |  |
| --- | --- | --- |
| 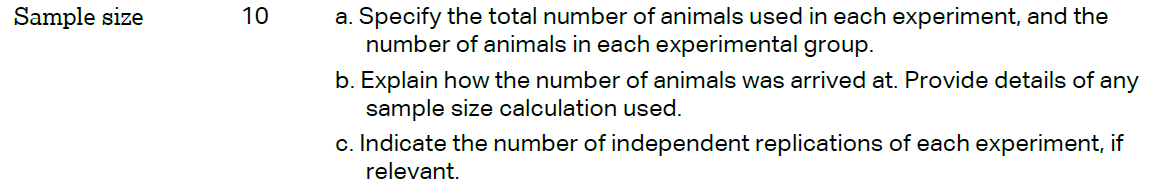 | Blood samples (n=10) were collected from wing vein at the 21 and 35 days of age without anticoagulant for serum separation. Collected samples were centrifugated at 4000 rpm for 5 min at 4°C to obtain clear sera for HI test against NDV and biochemical analysis.  Samples of 1 cm were taken from ileum (5 cm from Meckel’s diverticulum) and washed immediately with physiological saline (0.9% NaCl). Each sample was kept in an Eppendorf tube and immersed instantly in the liquid nitrogen.  Additionally, samples of 3 cm were obtained from jejunum (1 cm cut from the midpoint) were washed with saline and kept in 10% neutral buffered formalin for 24 h for histological examination. |  |
| 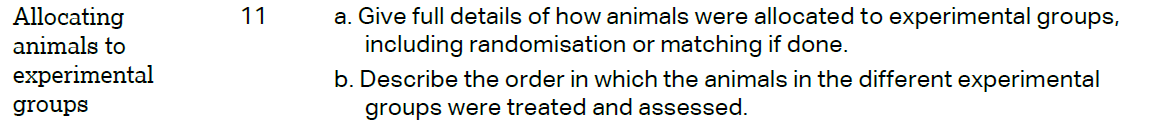 | The chicks were allocated into a control group 1 (CON, fed on a commercial basal diet), group 2 (LYZ70), group 3 (LYZ90), and group 4 (LYZ120) were fed on a commercial basal diet containing 70, 90, and 120g lysozyme 10%® (Nan Chang Lifeng Industry and Trading Co., Ltd., Jiangxi, China) per ton, respectively. |  |
| 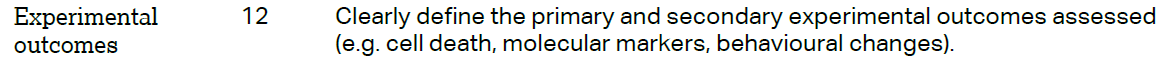 | The results of the current study showed that the exogenous lysozyme supplementation to the broiler chickens improved the bird growth performance and gut microbiota through enhancement of intestinal health that monitored by the molecular expression of non-specific immune and antioxidant genes. This study is considered a novel one that established the recommended dietary level of exogenous lysozyme supplementation at a dose 90g lysozyme® per ton of basal diet. |  |
| 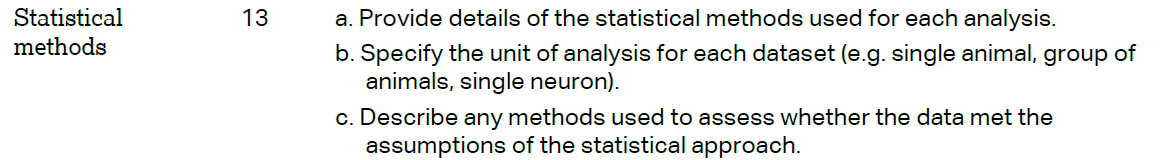 | The statistical measures were handled by the SPSS programming tool (IBM SPSS. 20®, Michigan, USA) using the One-way ANOVA followed by Duncan’s multiple range tests. The obtained data of HI assay, RT-PCR, and total intestinal bacterial counts were analysed by One-way ANOVA, Tukey’s multiple range tests by GraphPad Prism 5 (San Diego, CA, USA). All declarations of significance depended on p <0.05. |  |
| RESULTS |  |  |
| 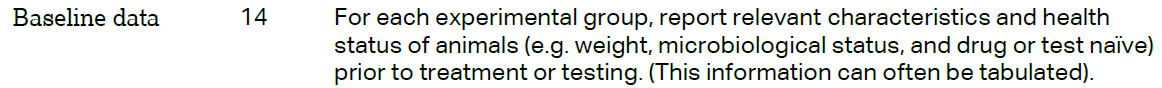 | The birds were received health from the hatchery with suitable performance and weight. |  |
| 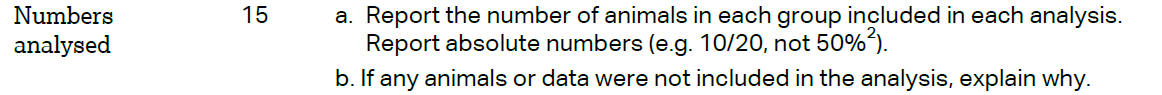 | Blood samples, intestinal samples for gene expression and intestinal samples for histological examination were subject for analysis (*n*=10).  All data were subjected to statistical analyses. |  |
| 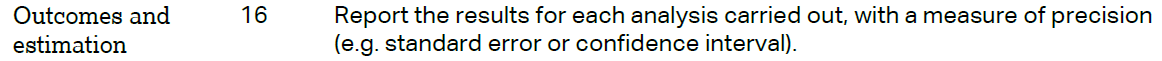 | This study is considered a novel one that established the recommended dietary level of exogenous lysozyme supplementation at a dose 90g lysozyme® per ton of basal diet. |  |
| 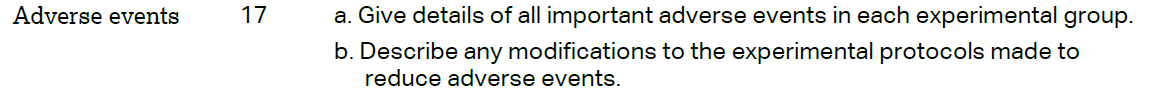 | Adverse effects were stated in LYZ120 group with significant increase in harmful intestinal bacteria with less beneficiary one. |  |
| DISCUSSION |  |  |
| 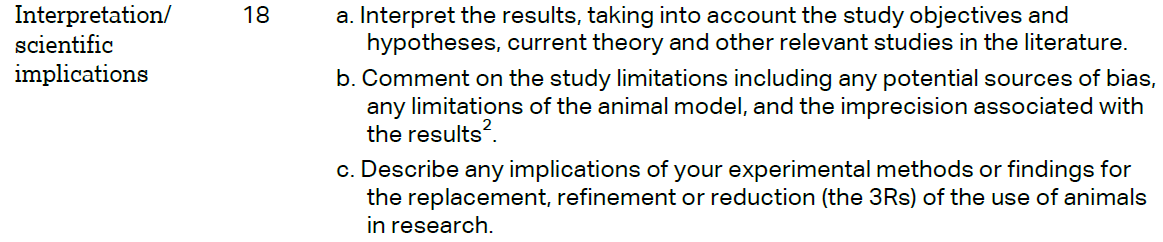 | The supplementation of Ross 308 birds with lysozyme especially LYZ90 improved the performance of birds with significant expression of INF-γ and antioxidant genes (SOD1 and GSH-Px). |  |
| 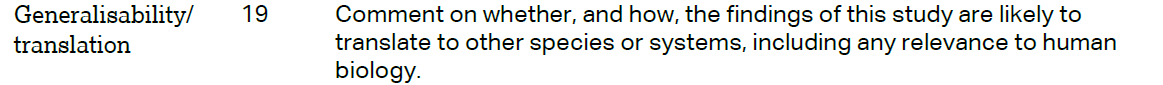 | The results of this study of great benefits for human consumption of antibiotic-free poultry meats.  Lysozyme may be used to improve the GIT of human and animals. |  |
| 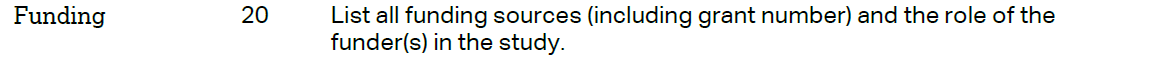 | | No finding organization for this study. |

Ibrahim, H.R., Yamada, M., Matsushita, K., Kobayashi, K., Kato, A., 1994. Enhanced bactericidal action of lysozyme to Escherichia coli by inserting a hydrophobic pentapeptide into its C terminus. J. Biol. Chem. 269, 5059–63.

Ivanovska, N., Georgieva, P., Barot-Ciorbaru, R., 1996. Correlation between inhibited alternative complement activity and the protective effect induced by Nocardia lysozyme digest (NLD) during Klebsiella pneumoniae infection in mice. Int. J. Immunopharmacol. 18, 515–9.

Lallès, J.-P., 2016. Microbiota-host interplay at the gut epithelial level, health and nutrition. J. Anim. Sci. Biotechnol. 7, 66. doi:10.1186/s40104-016-0123-7

Liu, D., Guo, Y., Wang, Z., Yuan, J., 2010. Exogenous lysozyme influences Clostridium perfringens colonization and intestinal barrier function in broiler chickens. Avian Pathol 39, 17–24. doi:10.1080/03079450903447404


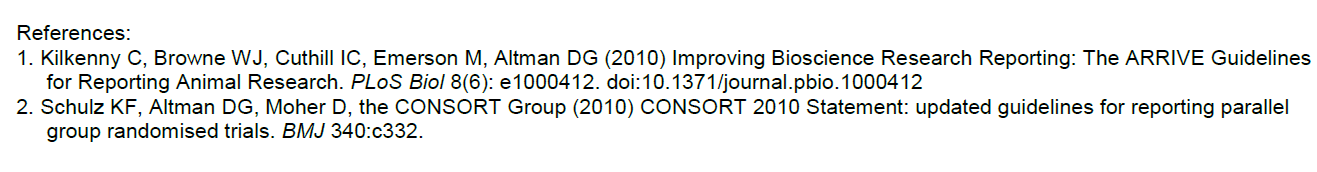

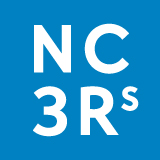

Supplement: S1 Table — (DOCX) [file pone.0185153.s002.docx]
